# Autologous Mesenchymal Stem Cell Treatment is Consistently Effective for the Treatment of Knee Osteoarthritis: The Results of a Systematic Review of Treatment and Comparison to a Placebo Group

**DOI:** 10.3390/medicines7080042

**Published:** 2020-07-24

**Authors:** Chadwick Prodromos, Susan Finkle, Tobias Rumschlag, John Lotus

**Affiliations:** Illinois Sportsmedicine and Orthopaedic Center, Glenview, IL 60025, USA; suef@ismoc.net (S.F.); tobyr@ismoc.net (T.R.); johnlotus21@gmail.com (J.L.)

**Keywords:** mesenchymal stem cell, autologous, bone marrow aspirate, adipose tissue, stem cell, knee osteoarthritis

## Abstract

**Background:** Numerous studies have used autologous mesenchymal stem cell injections (AMSCI) to treat osteoarthritis. We hypothesized that AMSCI is an effective osteoarthritis treatment with increasing efficacy at higher doses. **Methods:** We conducted a PubMed search for human clinical studies using AMSCI for the treatment of osteoarthritis (OA) and a second search for placebo arms of injectate OA treatment. Inclusion criteria included treatment outcomes ratings both pre-treatment and at least 6 months post-treatment. **Results:** 45 AMSCI cohorts from 34 studies met criteria. All AMSCI cohorts showed improvement at mean 15.3 months post-treatment. Mean WOMAC and VAS scores improved at 6-months and at final follow-up (*p* < 0.0001 for all). Scores > 2 years were also significant (WOMAC *p* = 0.001/VAS *p* = 0.004). Results greatly exceeded the minimal clinically important difference (MCID) at each time point. AMSCI improvement also substantially exceeded previously published 6-month placebo-treatment improvement. No dose–response relationship was seen. AMSCI cohorts showed continuing improvement ≥ 6 months, and continued upward at one year. Placebo scores were already trending downward by 6 months. **Conclusions:** AMSCI is a consistently significantly effective treatment for osteoarthritis. It should no longer be stated that data is insufficient to establish AMSCI efficacy for OA. Given its excellent safety profile, AMSCI should be widely used for the treatment of osteoarthritis.

## 1. Introduction

Osteoarthritis afflicts tens of millions of people worldwide, resulting in tremendous morbidity and economic costs [1]. The most common, non-surgical treatments include use of oral pain medication including non-steroidal anti-inflammatory drugs (NSAIDs) and opiates, and joint injections with corticosteroids or hyaluronic acid (HA). All of these options have serious safety and efficacy issues. NSAID use is associated with high toxicity rates [2]. HA has a variable effect, with low efficacy in many patients and a short (6-month) duration of effect [3]. Corticosteroid injections can damage cartilage [4,5], predispose to serious problems after a joint replacement [6,7,8,9,10,11], and have been shown to increase the incidence of joint replacement [4,10]. Therefore, there is an urgent need for a non-surgical treatment that is safe and more effective than these options.

The intra-articular injection of autologous mesenchymal stem cells (AMSCI) has been used in several studies for the treatment of knee osteoarthritis. AMSCIs are used in several forms including stromal vascular fraction (SVF), culture expanded adipose derived stem cells (ASCs), minimally manipulated fat graft (MM Fat), bone marrow aspirate (BMAC), and culture expanded bone marrow (BMSCs). A review of the literature has shown that they are completely safe in this application [12]. If effective, they would be a valuable tool for the treatment of arthritis. Based on reports in the literature and our own experience, we hypothesized that a comprehensive literature review would show that intra-articular AMSCI is an effective treatment for knee osteoarthritis and that the outcomes would be significantly better than a historical control group of placebo treated patients taken from placebo arms of other knee injection studies for treatment of osteoarthritis. We also hypothesized that there would be a positive dose–response relationship with improved outcomes with increasing dose of cells for treatment among the cohorts.

## 2. Materials and Methods

**Figure 1:**
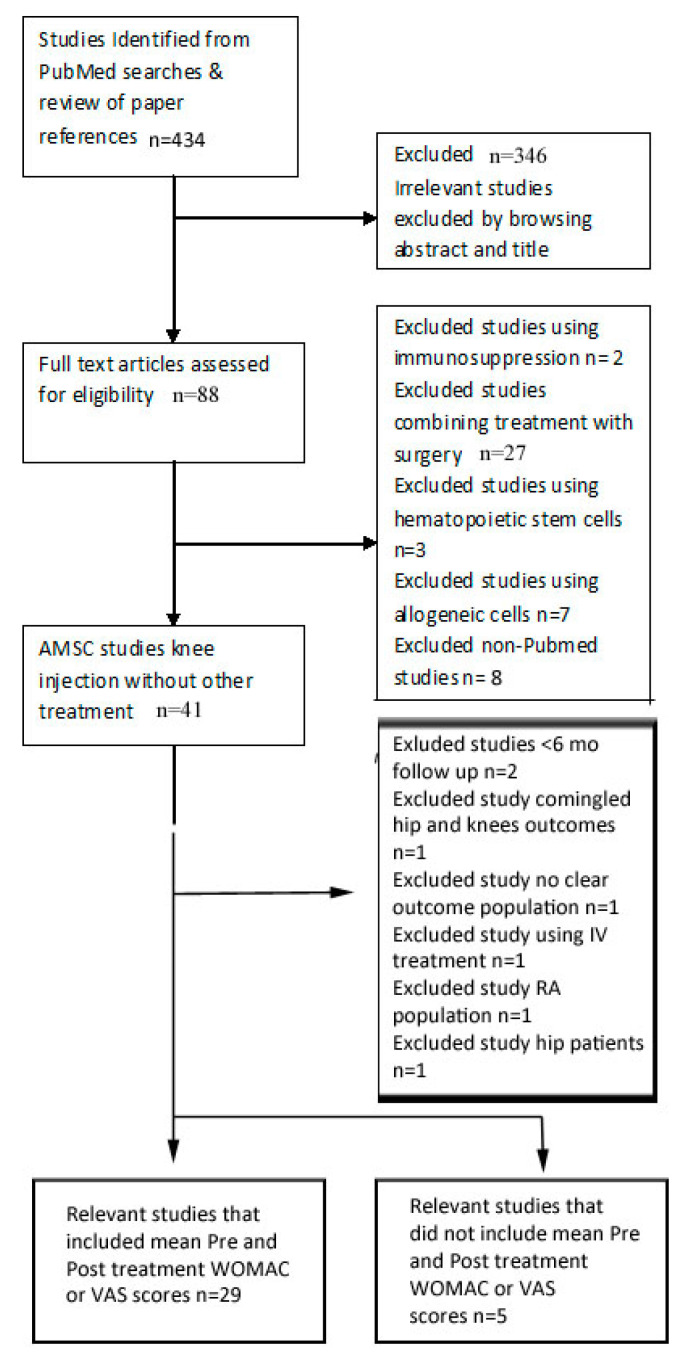
Flow diagram—study selection process.

We performed a comprehensive literature search using the PubMed database for the treatment of knee osteoarthritis with mesenchymal stem cells (MSCs) in humans, using the terms: “osteoarthritis/arthrosis”, “stem cell”, “mesenchymal stem cell”, “MSC”, “stromal vascular fraction”, “SVF”, “minimally manipulated fat”, “micro-fragmented fat”, “adipose-derived stem cell”, “bone marrow derived stem cell”, “cultured stem cell”, and “bone marrow aspirate”. In addition, we reviewed the references of the articles found this way to find other articles potentially matching our criteria. All papers found were read thoroughly, and papers were included in the study if they reported clinical outcome data pre-treatment and at least six months post-treatment. The included papers were separated into two groups, those that included pre- and post- mean Western Ontario and McMaster Universities Osteoarthritis Index (WOMAC) scores and/or pre- and post- mean visual analogue scale (VAS) scores of patient self-reported global pain to evaluate outcomes (Group 1), and those that used other evaluation outcome ratings (Group 2). Papers were excluded that combined stem cell treatment with surgery or immunosuppression, used hematopoietic or allogeneic stem cells, or did not use joint injection as the treatment approach (see Figure 1).

We performed a second comprehensive literature search of PubMed for randomized clinical trials in adult humans of therapy for osteoarthritis of the knee which included a single injection or up to three injections of ‘normal’ or physiological saline (0.9% NaCl) or phosphate-buffered saline (PBS) as a placebo cohort. The search was performed for the keywords “knee”, “placebo”, and “arthritis”, as well as any one of the following keywords for an intra-articular injective therapy for osteoarthritis: “Adant”, “Arthrease”, “Arthrum”, “Artz”, “Artzal”, “Biohy”, “Clodronate”, “cortisone”, “Durolane”, “Euflexxa”, “Fermathron”, “Gel-200”, “Gel-one”, “Gelsyn-3”, “Genvisc”, “Go-on”, “Hya-ject”, “Hya-joint”, “hyalgan”, “hyaluronate, hyaluronic acid”, “Hylan g-f 20”, “Hymovis”, “Interleukin-1 receptor antagonist”, “lmwf-5a”, “Monovisc”, “Nasha”, “Nrd-101”, “Nuflexxa”, “Orthovisc”, “Ostenil”, “platelet-rich plasma”, “Replasyn”, “Slm-10”, “sodium hyaluronate”, “steroid”, “steroidal”, “Structovial”, “Sunevyl”, “Supartz”, “Suplasyn”, “Synject”, “Synovial”, “Synvisc”, “Synvisc-one”, “tgf-ß1-expressing chondrocytes”, “triamcinolone acetonide”, “Variofill”, or “Zeel compositum”. Studies were discarded if they were not written in English and if the injective therapy was paired with another intervention, including physical therapy programs and surgical procedures. Studies were excluded if they did not include at least a 3-month follow-up. Similarly to the AMSCI studies, only studies that included WOMAC and/or VAS pre- and post-treatment scores were included. This group was labeled as Group 3.

Group 1 papers were evaluated, and the follow-up duration and WOMAC and VAS scores pre- and post-treatment were noted. Mean scores were calculated for pre-treatment, at 6 months post-treatment, maximum change post-treatment, and final change post-treatment. For the WOMAC score, all papers were converted to a 0–96-point scale, and for the VAS, all scores were converted to a 0–100 point scale. All other reported result intervals were noted. Given the variability in study protocols, each study was weighted equally in calculating these means.

Group 2 papers were evaluated for the outcome measures that were used and the outcomes that were reported.

All the included papers were categorized based on the type of MSC preparation used. Each subgroup was individually analyzed.

Group 3 papers were evaluated in the same manner as Group 1 papers, and the follow-up duration and WOMAC and VAS scores pre- and post-treatment were noted. Mean scores were calculated for pre-treatment, at 3 months post-treatment, and at 6 months post-treatment, where available. Peak time of effect was noted. WOMAC scores were converted to a 0–96-point scale and VAS scores were converted to a 0–100 point scale as needed. All other reported result intervals were noted. Given the variability in study protocols each study was weighted equally in calculating these means.

## 3. Results

Our search yielded 34 studies published in peer-reviewed journals that examined the injection of autologous AMSCI for the treatment of knee osteoarthritis and met our criteria for rating evaluation. Of these papers, 29 studies included mean WOMAC and/or VAS scores (Group 1) and 5 did not (Group 2) (see Table 1 and Table 2). Our second search found 18 studies that had placebo-controlled arms, which were included in Group 3 (see Table 3).

### 3.1. Group 1

The 29 studies in Group 1 included 1063 treated knees. Six of the studies included two or more arms with different AMSCI treatments, resulting in 38 treatment arm subgroups included in this analysis. Twelve of the subgroups were treated with ASCs, 7 were treated with SVF, 7 were treated with BMAC, 6 were treated with BMSCs, and 4 were treated with MM Fat, and 2 were treated with BMAC & MM Fat. The number of cells used in each treatment is listed in Table 1. Follow-up time after treatment ranged from 6 months to 5 years, with a mean time of 14.4 months for the final follow-up. All studies reported significant improvement after treatment. Group 1 was broken into 6 subgroups based on the type of AMSCI used in the treatment. The outcomes for each subgroup are also reported in Table 1.

Twenty-six of the study subgroups included WOMAC scores, with 6-month follow-up WOMAC scores being available for 22 study subgroups. The mean pre-treatment WOMAC score of these studies was 45.1 (scale 96 to 0, 0 best). The mean WOMAC score decreased at the six-month follow-up to 25.9 and to 20.3 at the final follow-up point, for a total change in score of 23.0 and 25.2, respectively. Comparison of means was performed between pre-treatment scores and post-treatment scores at 6 months and final follow-up, and were significantly different at all follow-up points (*p* < 0.0001 for all).

An AOSSM Outcome Task Force led by Irrgang [49] reported that the minimal clinically important difference (MCID) for the WOMAC total score for knee osteoarthritis was 11.5 on a 100-point scale, which is the equivalent of 11.0 on a 96-point scale. The mean WOMAC improvements at 6 months and final follow-up were more than twice those with MCID. The improvement in WOMAC scores exceeded the MCID in 25 of 26 subgroups. The paper that fell below the MCID [28] reported a change in the final score of 9.5, close to the MCID of 11.

Thirty-two study subgroups included VAS scores. Six-month VAS scores were available for 27 study subgroups. The mean pre-treatment VAS score was 56.2 (0 to 100, 0 best). The mean VAS score decreased to 29.6 at six months and 27.1 at the final follow-up point, for a total change in score of 28.7 and 28.7. Comparison of means was performed between pre-treatment scores and post-treatment scores at 6 months and final follow-up and was significantly different from pre-treatment to all follow points (*p* < 0.0001 for all).

Tubach [50] reported that the MCID for knee osteoarthritis had a VAS pain score of 19.9. The mean VAS improvement was 29.3 at six months and 28.7 at the final follow-up. BMAC, however, showed lower levels of improvement on average than the other treatments. Only one study arm in the BMAC group [16] exceeded the MCID for VAS, resulting in the BMAC treatment group being the only group whose mean VAS improvement was less than the MCID. If BMAC results are excluded, then the improvement in VAS scores exceeded the VAS MCID in 22 of 26 subgroups at either 6-month or final follow-up. Two of the subgroups [26,29] in which the VAS did not exceed the MCID had WOMAC scores that did exceed the MCID. One of these had a VAS score of 19.7, very close to the 19.9 for MCID. The remaining two subgroups [14,17] did not report WOMAC scores and were both minimally manipulated fat (one with additional BMAC).

**Figure 2:**
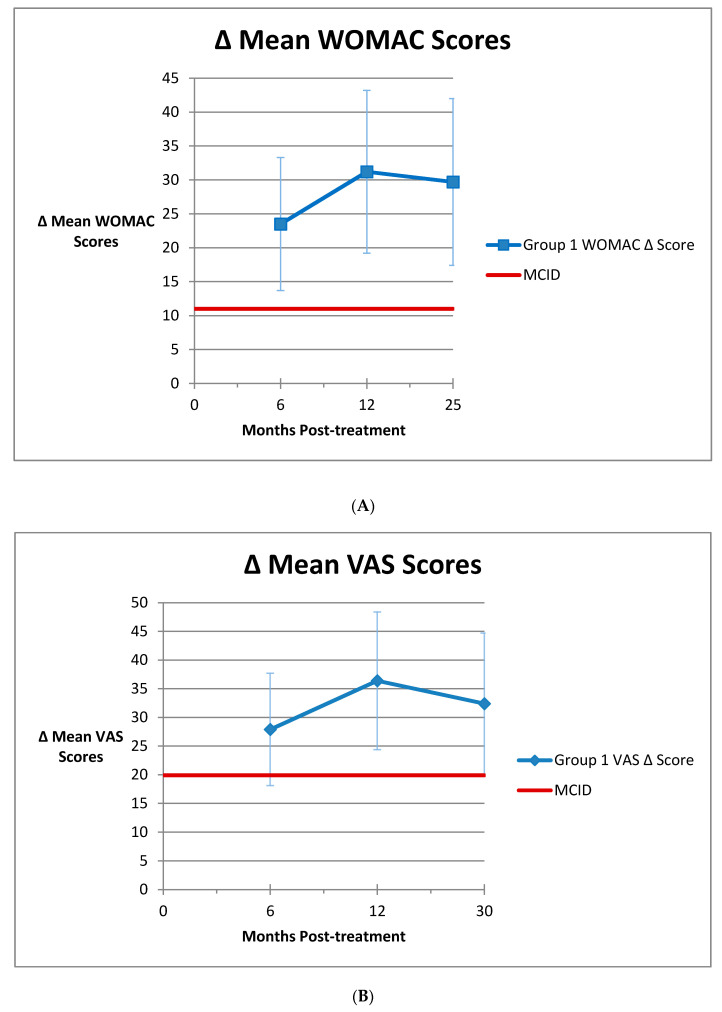
(**A**) Intermediate and long-term mean improvement in WOMAC scores starting from 6 months post-treatment. Vertical bars indicate 95% confidence interval. (**B**) Intermediate and long-term mean improvement in VAS scores starting from 6 months post-treatment. Vertical bars indicate 95% confidence interval.

Six study arms of four studies in Group 1 reported changes in WOMAC scores for 6 months, 1 year and some point longer than 1 year [26,30,37,43]. The long-term follow-up was a mean of 25 months post-treatment for the WOMAC scores. Figure 2A shows the scores for the 6 studies with intermediate and long-term WOMAC scores. The MCID is indicated in this figure. Nine study arms of seven Group 1 studies reported change in VAS scores for all three points [21,26,30,31,35,40,41,42]. The long-term follow-up was a mean of 29 months post-treatment for the VAS scores. The mean of these points is shown in Figure 2B. The VAS MCID is indicated in this figure. A comparison of means between the pre-treatment scores and the long term follow-up point showed that the scores were statistically significantly improved (*p* = 0.001 for WOMAC, *p* = 0.004 for VAS).

### 3.2. Group 2

The five studies in Group 2 had seven subgroups which included 193 joints and used a variety of other outcome scores (See Table 2) Lamo-Espinosa [37] used both WOMAC and VAS scores, but reported only medians, so the numbers could not be included with the Group 1 papers. The studies from Al-Najar [29], Bastos [30] and Gibbs [45] all reported KOOS scores. Roos [51] reported the MCID for KOOS scores as 10 for each subsection. All the reported KOOS scores were above the MCID. The study from Centeno [31] used a Likert scale. In all cases, the reported scores improved significantly from baseline to the final follow-up point.

### 3.3. Group 3

There were 18 studies, one with two placebo arms, in Group 3, resulting in 19 groups with a total of 1793 patients (see Table 3). Sixteen reported WOMAC scores and 10 reported VAS scores. Only one study [52] reported results past 6 months. The mean change in WOMAC score was 9.2 at 3 months and 10.5 at 6 months. The mean change in VAS scores was 12.9 at 3 months and 13.6 at 6 months. The mean change in WOMAC score was below the MCID at 3 and 6 months. The mean change in the VAS scores was substantially below the MCID at all time points. For studies that reported at least 2 follow-up times, the maximal improvement time point was noted. Eleven studies that reported WOMAC scores and 10 that reported VAS scores fit this criterion. The maximal improvement in these studies was reached early with the maximal effect at a mean of 10.0 weeks for WOMAC scores and 8.4 weeks for VAS scores. Two studies reported no improvement [53,54]. Comparison of means was performed between pre-treatment WOMAC and VAS scores and scores at 3 months and 6 months post-treatment. WOMAC score were statistically different at 3 months but not at 6 months (3 mo *p* = 0.04, 6 mo *p* = 0.07). VAS scores were not statistically different from pre-treatment to post-treatment at either time point (3 mo *p* = 0.11, 6 mo *p* = 0.10). Comparison of means was performed comparing the change in mean at 6 months between Group 1 and Group 3. The means of the two groups were statistically different for both WOMAC (*p* < 0.0001) and VAS (*p* = 0.003).

**Figure 3:**
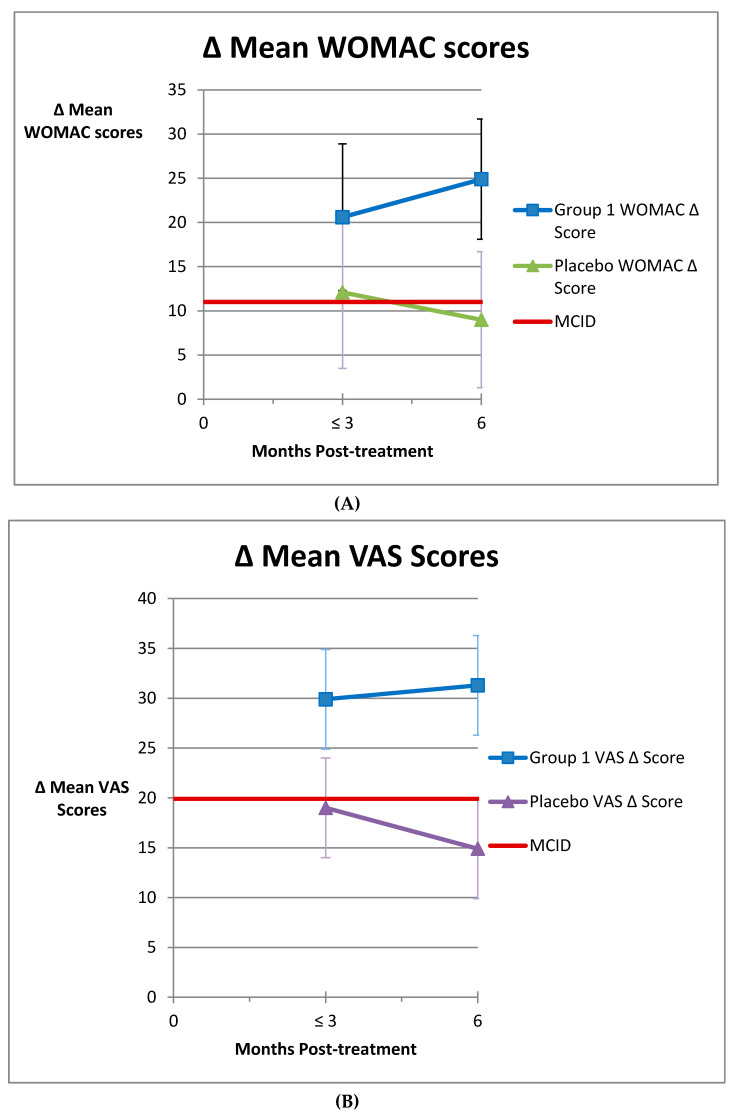
(**A**) Comparison of maximum mean WOMAC improvement before 3 months post-treatment compared to 6 months post-treatment for Group 1 and placebo patients. Vertical bars indicate 95% confidence interval. Comparison of means between Group 1 and placebo was *p* = 0.02 at ≤3 months and *p* =< 0.0001 at 6 months. (**B**) Comparison of maximum mean VAS improvement before 3 months post-treatment compared to 6 months post-treatment for Group 1 and placebo patients. Vertical bars indicate 95% confidence interval. Comparison of means between Group 1 and placebo was *p* =0.02 at ≤3 months and *p* = 0.001 at 6 months.

The mean WOMAC and mean VAS scores for Group 1 and Group 3 studies that reported a 6-month score and at least 1 endpoint prior to 6 months are shown in Figure 3A and Figure 3B, respectively. There are 16 Group 1 and 9 Group 3 subgroups that reported WOMAC scores and 18 Group 1 and 9 Group 3 subgroups that reported VAS scores at and prior to 6 months included in these figures. The MCID for each score is also shown on these figures. The Group 1 WOMAC scores are substantially above the MCID and rising while the Group 3 scores start above the MCID and decline below that level by 6 months. Comparison of means between the Group 1 and placebo WOMAC scores is significantly different at all points (*p* = 0.02 at ≤3 months, *p* < 0.0001 at 6 months). The VAS scores are similar, except that the Group 3 scores are never above the MCID. Comparison of means between the Group 1 and placebo VAS scores is significantly different at all points (*p* = 0.02 at ≤3 months, *p* = 0.001 at 6 months).

A comparison was made using Groups 1 and 2 based on the type of AMSCI used. BMAC, BMAC/MM Fat and MM Fat groups were excluded because of low reporting of WOMAC scores in these groups with only three reported results in the MM Fat group, two in the BMAC group and none in the BMAC+ MM Fat group. For the remaining three groups (ASCs, BMSCs, and SVF), a MCID ratio was calculated for each study at 6 months and at final follow-up. For Group 1 studies and the Group 2 study that used WOMAC scores, the MCID ratio was calculated as the reported WOMAC score divided by the WOMAC MCID of 11.0. For the Group 2 studies that reported KOOS scores, the MCID ratio was the sum of the scores from the five subgroups divided by 5 and then divided by the KOOS MCID of 10. The MCID ratios can be found in Table 4. There were 11 ASC studies, 10 BMSC studies and 6 SVF studies in this comparison. The mean MCID ratios at final follow-up were 2.5 for ASCs, 2.1 for BMSCs, and 3.0 for SVF. Although the BMSC results were slightly lower than the other two groups, a comparison of means showed no significant differences between the groups.

**Figure 4:**
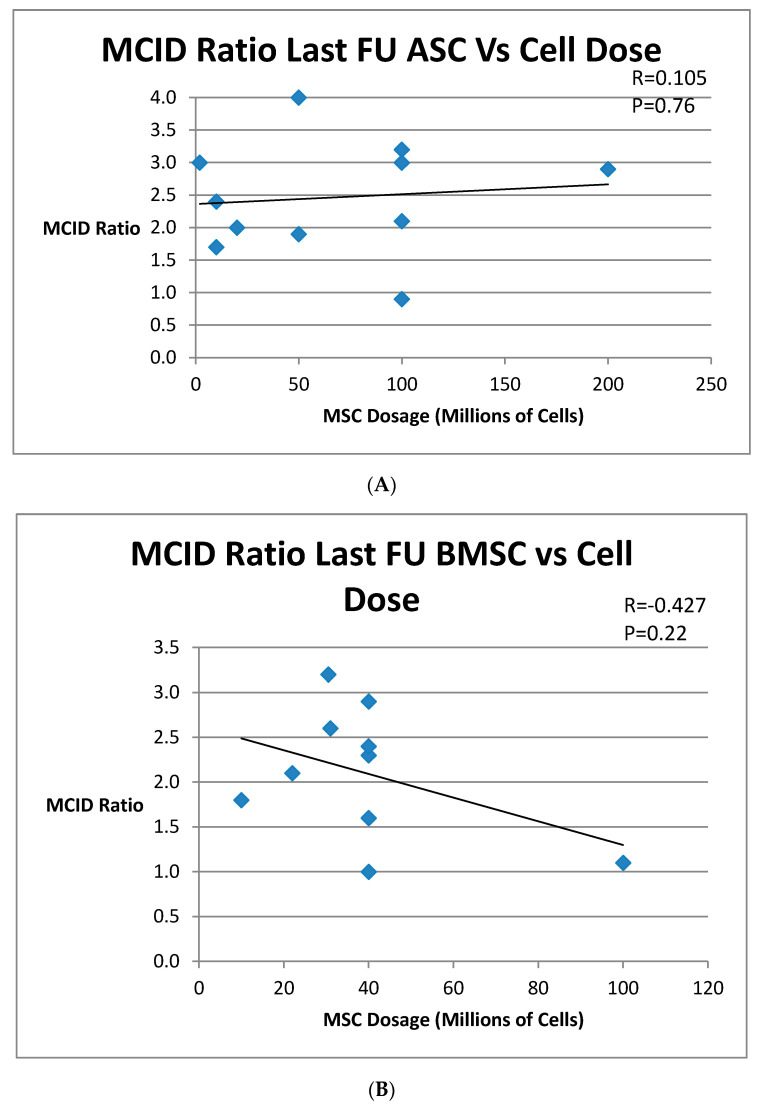

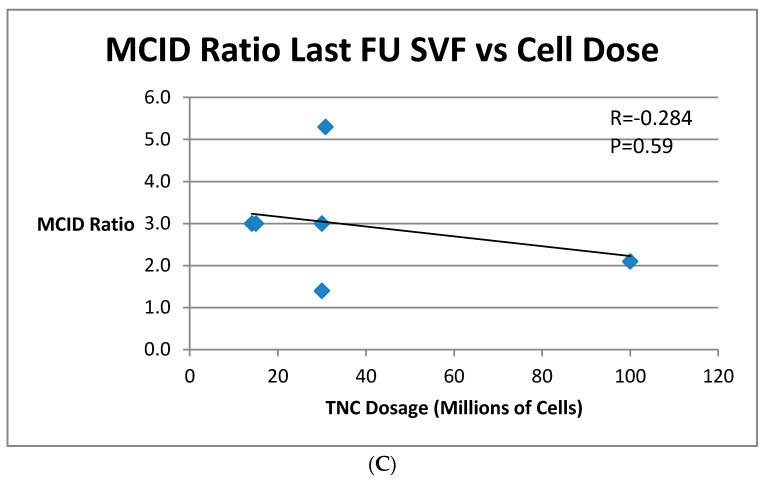
(**A**) MCID Ratio at Last Follow-up for Cultured ASC vs MSC Dose. (**B**) MCID Ratio at Last Follow-up for Cultured BMSC vs MSC Dose. (**C**) MCID Ratio at Last Follow-up for SVF vs TNC Dose.

A comparison was performed to see if a dose–response relationship existed between numbers of cells and outcome parameters. For each of the three groups, ASCs BMSCs, and SVF, the MCID ratio at final follow-up was compared to the total number of cells used in treatment. For ASC and BMSC groups, the total number of cells was based on the reported MSC cells. For the SVF group, the reported total nucleated cells (TNCs) were used. A regression analysis was performed. The results are graphed in Figure 4A–C. No statistically significant dose–response relationship was found, and the p values were not significant for any of these groups. Although there was a trend toward decreasing efficacy with greater stem cell dose for both BMSCs and SVF, ASCs showed a slightly increasing efficacy. However, none of these trends were statistically significant.

## 4. Discussion

This is the first paper to examine the spectrum of autologous mesenchymal stem cell treatment in the treatment of osteoarthritis for efficacy. It is the first to show consistent clinically important efficacy from these treatments. It is also the first to examine dose–response curves for these treatments and demonstrate the lack of same so far.

All 26 of the Group 1 subgroups which included WOMAC scores showed improvement from pre-treatment scores to final follow-up. These improvements were statistically significant at all time points. Treatment with AMSCI produced, on average, strong improvement in WOMAC scores that continued to improve over time, with one year follow-up showing greater improvement than 6-month follow-up.

In contrast, the Group 3 placebo WOMAC mean change peaked before 3 months and was 9.0 at 6 months and falling. Although the scores were both statistically improved and slightly above the MCID initially, showing some placebo or saline effect, the results fell off quickly and by 6 months were well below the MCID and clinically insignificant. These results demonstrate that the placebo effect peaks early and is not large. The effects of treatment seen in the Group 1 results are larger, more sustained, and continue to rise over time, showing a definite positive effect of treatment well beyond the placebo effects of saline injection.

Similarly, all 23 Group 1 study subgroups that included VAS scores showed improvement from pre-treatment to all short, intermediate, and long term follow-up points. These improvements were statistically significant at all time points.

The Group 3 placebo studies had a mean change in VAS scores substantially below the MCID at all time points. Only three of the studies exceeded the MCID at any point in time. VAS scores peaked early (about 2 months) and the results declined from there. The follow-up results were not statistically different from the pre-treatment scores at any point. Similarly to the WOMAC scores, the change in VAS scores demonstrate a small placebo effect and a much larger improvement in Group 1, which indicates significant improvement from treatment with AMSCI.

In the case of both the WOMAC and VAS scores and as seen in Figure 2A,B, the Group 1 improvements continue out to the ends of the studies (mean 25 months for WOMAC, 30 months for VAS), and although they begin to decline eventually, they remain substantially above the MCIDs. Again, these differences demonstrate that the improvements resulting from AMSCI are not due to a placebo effect, but instead are actual results of treatment. The duration of clinical improvement is an important factor in treatment with AMSCI. The mean duration of follow-up in the studies here was 14.2 months, with a range between 6 months and 5 years. All but one study showed clinically significant improvement out to the end of the follow-up time period. HA studies in general only pursue follow-up to 6 months, and even at six months, results are not as good as with AMSCI treatment. [3] Placebo studies are usually 6 months or less and show maximal results 2 to 3 months post-treatment, with improvement declining rapidly after that. Corticosteroid injections offer good short term relief, but the effects usually only last 4 to 6 weeks [70]. The short-term results of treatment with HA and corticosteroids require more frequent treatment, thereby increasing the risks of adverse events from treatment. Corticosteroids have been shown to accelerate the progression of osteoarthritis [4,5] and predispose to serious problems after joint replacement [6,7,8,9,10,11]. This study demonstrates that a single treatment of AMSCI can produce significant clinical improvement that can last at least 2 years. Other results from our center showed significantly improved results out to 2 years with a trend toward further improvement from the one year to the two year follow-up, after a single treatment with fat, bone marrow and PRP [71]. Our hypothesis was thus validated regarding comparison to placebo.

Of the six different types of AMSCI treatments in this study, three of them (MM fat, BMAC, and BMAC + MMF) had too few cohorts with WOMAC or KOOS scores to allow meaningful comparison. In the other three groups (SVF, ASCs, and BMSCs), the change in scores was very similar between the groups at both 6 months and final follow-up.

It was hypothesized that there would be a positive relationship between cell number and improved outcome. However, no dose–response relationship between cell dose and outcome was found, refuting this part of our hypothesis. This was true for both MSCs and SVF, which measures TNC. This universally beneficial effect of AMSCI across widely varying doses indicates that there may be a threshold effect demonstrated, but not a dose–response relationship. A meta-analysis of mesenchymal stem cells from Cui [72] also found no dose–response association. A dose–response relationship, however, has been seen in other biologics studies [29,45,73]. A study by Garza [45] found a dose–response relationship for patients treated with SVF, with a statistically positive relationship with greater improvement in knee arthritis with a larger stem cell dose. However, this relationship is for a percent improvement between two cohorts with different WOMAC starting points. The absolute change in WOMAC between the cohorts did not differ significantly.

The presences or absence of a dose–response curve is a matter of some importance, because increased dosing of cultured cells engenders greater cost. Increased dosing of stromal vascular fraction or bone marrow aspirate increases the time and discomfort of the procedure. If smaller doses have equal efficacy to larger ones, the cost and difficulty of AMSCI procedures could be better controlled. The addition of data from future studies should help to settle this question and generate optimal dosing regimens and perhaps identify a minimally effective cell dose.

Limitations of this study are the limited number of cohorts available for analysis. However, the fact that every one of the 45 treatment subgroups showed clinical efficacy is more than a sufficient number to validate our hypothesis of consistent AMSCI efficacy. The heterogeneity of the studies also limited our ability to compare treatment types.

These results show that AMSCI treatment has, arguably, the greatest proven efficacy of any known treatment for osteoarthritis. The treatment is longer lasting than other non-surgical treatments and has the best safety profile. Joint replacement carries significant morbidity and mortality, and often produces poorer outcomes than expected by patients [74]. HA and corticosteroids, as discussed above, have much shorter duration of effect and often significant adverse effects. Oral analgesics have limited duration of effect, do nothing to mitigate the disease process, and have frequent morbidity and mortality issues. NSAID drugs interfere with joint healing and are quite toxic, with an estimated 16,000 deaths annually in the USA alone secondary to their use [2]. Opiate use for treatment of arthritis has resulted in addiction and overdose sequela, increased risks of falls with resulting fractures, and overall increased mortality without providing good relief of symptoms [75,76,77,78].

Up until now, discussions of stem cell treatments have generally begun with a disclaimer that they are certainly “promising”, but that there is “insufficient data” to determine if they are effective or safe. We believe that this study conclusively refutes this agnosticism and changes that narrative. In fact, the results reported here show that there is more than enough data to conclude that autologous mesenchymal stem cell treatment, while not a “cure”, is clearly consistently significantly effective for OA. Our companion study [12] shows that it is also completely safe, with no significant adverse events ever having been reported in the PubMed indexed literature for injections for arthritis. While the exact degree of clinical efficacy to be expected from AMSCI treatment for osteoarthritis is still being determined, and much work obviously needs to be done, clinical efficacy was seen in all AMSC treatment studies reported here. Further understanding of optimal treatment paradigms can only come from more clinical studies. Restricting clinical AMSCI use because of supposed, but not actual, concerns for safety or lack of efficacy has the effect of inhibiting the ability to continue to improve clinical efficacy through unwarranted restriction of this important clinical research. It also results in denial of this safe and effective treatment for patients who could benefit from it by physicians who are misled by this false narrative. This in turn results in substantial morbidity, mortality, and economic cost from the complications of surgery and treatment with non-steroidal anti-inflammatory drugs [2], steroid injections [79], and other treatments, which could have been avoided if stem cell treatments were widely available.

## 5. Conclusions

This study demonstrates that autologous mesenchymal stem cell injection provides consistent clinically important improvement in OA disease parameters, and that both the magnitude and the duration of improvement far exceed any improvement that may be seen from a placebo effect and is well above the MCID. Furthermore, the duration of efficacy out to two years from a single treatment greatly exceeds that of any other nonsurgical treatment or any possible placebo effect. Particularly in light of its excellent safety profile and the absence of other safe effective treatments for osteoarthritis, AMSCI treatment should be widely adopted for the treatment of osteoarthritis.

## Figures and Tables

**Table 1:** Included MSC studies—includes all Group 1 and Group 2 studies, broken down by MSC type.

					WOMAC Scores					VAS Scores				
Author/Year/ Cohort	Cell Type	Dosage	Study FU Length (Mo)	# of Joints	Pre-treatment	6 mo	Δ 6 mo	Final FU	Δ Final FU	Pre- treatment	6 mo	Δ 6 mo	Final FU	Δ Final FU
Anz 2020 [13]	BMAC	7 mL BMAC	12	45	35.3	19.4	15.9	19.4	15.9	NA	NA	NA	NA	NA
Centeno 2014 BMAC [14]	BMAC	90 mL BMAC	10	220	NA	NA	NA	NA	NA	40.0	NA	NA	26.0	14.0
Centeno 2015 low dose [15]	BMAC	<4E8 TNC	11	67	NA	NA	NA	NA	NA	40.0	NA	NA	31.0	9.0
Centeno 2015 high dose [15]	BMAC	>4E8 TNC	11	49	NA	NA	NA	NA	NA	31.0	NA	NA	17.0	14.0
Garay-Mendoa 2018 [16]	BMAC	670E6 BM mononuclear cells	6	26	35.9	7.9	28.0	7.9	28.0	52.7	9.2	42.8	9.2	42.8
Mautner 2018 BMAC [17]	BMAC	8ml BMAC	6	58	NA	NA	NA	NA	NA	39	25	14	25	14
Shapiro 2016 [18]	BMAC	34E3 MSCs	6	25	NA	NA	NA	NA	NA	31.0	15.0	11.0	15.0	11.0
**BMAC Means**			**8.3**		**35.6**	**13.7**	**22.0**	**13.7**	**22.0**	**39.0**	**16.4**	**22.6**	**20.5**	**17.5**
**Total # of Studies 6/Subgroups 7**		**Total # of Joints**		**490**										
Centeno 2014 BMAC/Fat [14]	BMAC & MM Fat	90 mL BMAC, 10 mL MM fat	11	103	NA	NA	NA	NA	NA	43.0	NA	NA	30.0	13.0
Kim 2014 [19]	BMAC & MM Fat	7 mL BMAC, 10 mL MM fat	12	75	NA	NA	NA	NA	NA	70.0	35.0	35.0	33.0	37.0
**BMAC & MM Fat Means**			**9.0**		**NA**	**NA**	**NA**	**NA**	**NA**	**56.5**	**35.0**	**35.0**	**31.5**	**25.0**
**Total # of Studies 2/Subgroups 2**		**Total # of Joints**		**178**										
Dall’Oca 2019 [20]	MM Fat	5–10 mL microfragmented fat	6	6	36.3	19.8	16.5	19.8	16.5	46.0	15.0	31.0	15.0	31.0
Hudetz 2017 [21,22]	MM Fat	4–15 mL MM fat	24	17	NA	NA	NA	NA	NA	39.4	11.7	27.7	5.5	33.9
Mautner 2018 MM Fat [17]	MM Fat	9 mL MM Fat	6	48	NA	NA	NA	NA	NA	43.0	28.0	15.0	28.0	15.0
Pintat 2017 [23]	MM Fat	12 mL lipoaspirate	12	18	34.3	15.7	18.6	14.1	20.2	NA	NA	NA	NA	NA
Roato 2018 [24]	MM Fat	35 mL MM fat	18	20	45.9	15.8	30.1	13.0	32.9	70.5	30.1	40.4	33.4	37.1
**MM fat Means**			**13.2**		**38.81**	**17.1**	**21.7**	**15.6**	**23.2**	**49.7**	**21.2**	**28.5**	**20.5**	**29.3**
**Total # of Studies 5/Subgroups 5**		**Total # of Joints**		**109**										
Freitag 2019 1 inj [25]	ASCs	100E6 MSC × 1inj	12	9	38.8	16.3	22.5	15.4	23.4	67.0	29.0	38.0	26.0	41.0
Freitag 2019 2 inj [25]	ASCs	100E6 MSC × 2 inj	12	10	43.8	26.7	17.1	12.2	31.6	65.0	43.0	22.0	23.0	42.0
Jo 2017 low dose [26]	ASCs	10E6 MSCs	24	3	43.3	25.3	18.0	17.0	26.3	70.0	48.3	21.7	40.0	30.0
Jo 2017 medium dose [26]	ASCs	50E6 MSCs	24	3	69.0	48.5	20.5	25.1	43.9	78.3	67.0	11.3	66.0	12.3
Jo 2017 high dose [26]	ASCs	100E6 MSCs	24	12	54.2	32.8	21.4	19.0	35.2	79.6	44.2	35.4	45.8	33.8
Lee 2019 [27]	ASCs	100E6 MSCs	6	12	60.0	26.7	33.3	26.7	33.3	68.0	34.0	34.0	34.0	34.0
Lu 2019 [28]	ASCs	50E6 MSCs × 2inj	12	46	30.8	21.7	9.1	21.4	9.5	53.9	29.3	24.6	28.1	25.8
Pers 2016 low dose [29]	ASCs	2E6 MSCs	6	6	60.4	27.2	33.2	27.2	33.2	77.0	35.8	41.2	35.8	41.2
Pers 2016 medium dose [29]	ASCs	10E6 MSCs	6	6	41.9	22.8	19.1	22.8	19.1	63.7	36.7	27.0	36.7	27.0
Pers 2016 high dose [29]	ASCs	50E6 MSCs	6	6	35.8	14.8	21.0	14.8	21.0	43.7	24.0	19.7	24.0	19.7
Song 2018 [30]	ASCs	20E6 MSCs	24	14	34.8	20.4	36.8	12.4	22.4	49.4	26.2	23.2	31.7	17.7
Spasovski 2017 [31]	ASCs	0.5–1E7 MSCs	18	11	NA	NA	NA	NA	NA	54.5	9.3	45.2	9.1	45.4
**ASCs Means**			**14.5**		**46.6**	**25.7**	**22.9**	**19.5**	**27.2**	**64.2**	**35.6**	**28.6**	**33.3**	**30.8**
**Total # of Studies 7/Subgroups 12**		**Total # of Joints**		**138**										
Al-Najar 2017 [32]	BMSCs	30.5E6 MSCs	6	13	see Table 2									
Bastos 2018 MSCs [33]	BMSCs	40E6 MSCs	12	9	see Table 2									
Bastos 2018 MSCs + PRP [33]	BMSCs + PRP	40E6 MSCs + PRP	12	9	see Table 2									
Centeno 2011 [34]	BMSCs	MSCs cultured to 2-7th passage	11.3	135	see Table 2									
Davatchi 2016 [35]	BMSCs	8–9E6 MSC	60	4	NA	NA	NA	NA	NA	86.3	52.5	33.8	34.0	52.3
Emadedin 2012 [36]	BMSCs	20–24E6 MSC	12	6	67.1	41.9	25.1	43.6	23.5	57.0	10.0	47.0	11.6	45.4
Emadedin 2015 [37]	BMSCs	0.5E6 MSCs/kg bodyweight	30	6	69.8	43.7	26.1	41.7	28.1	NA	NA	NA	NA	NA
Emadedin 2018 [38]	BMSCs	40E6 MSCs	6	19	NA	NA	25.7	NA	25.7	NA	NA	20.8	NA	20.8
Lamo-Espinosa 2018 low dose [39]	BMSCs	10E6 MSC	48	10	see Table 2									
Lamo-Espinosa 2018 high dose [39]	BMSCs	100E6 MSC	48	10	see Table 2									
Orozco 2013/14 [40,41]	BMSCs	40E6 MSCs	24	12	18.7	NA	NA	7.2	11.5	46.9	24.8	22.1	17.0	29.9
Soler 2016 [42]	BMSCs	40E6 MSCs	12	15	26.5	NA	NA	9.0	17.5	51.3	21.0	30.3	19.0	32.3
**Cultured BMD- MESC Means**			**23.4**		**45.5**	**42.8**	**25.6**	**25.4**	**21.3**	**60.4**	**27.1**	**30.8**	**20.4**	**36.1**
**Total # of Studies 10/Subgroups 12**		**Total # of Joints**		**248**										
Bansal 2017 [43]	SVF	100E6 TNC	24	10	64.0	46.0	18.0	41.0	23.0	NA	NA	NA	NA	NA
Fodor 2016 [44]	SVF	14.1E6 TNC	12	8	32.9	NA	NA	9.4	32.5	59.0	NA	NA	20.0	39.0
Garza 2020 low dose [45]	SVF	15E6 TNC	12	13	54.0	22.8	31.2	20.9	33.1	NA	NA	NA	NA	NA
Garza 2020 high dose [45]	SVF	30E6 TNC	12	13	45.2	19.2	26.0	12.7	32.5	NA	NA	NA	NA	NA
Gibbs 2015 [46]	SVF	11.5E6–50E6 TNC	12	7	see Table 2									
Hong 2019 [47]	SVF	7.45E6 TNC	12	16	NA	NA	NA	NA	NA	53.8	16.9	36.9	21.9	31.9
Yokota 2017 [48]	SVF	30E6 TNC	6	26	49.6	33.8	15.8	33.8	15.8	72.7	49.2	23.5	49.2	23.5
**SVF Means**			**12.9**		**49.1**	**30.5**	**22.8**	**23.6**	**27.4**	**61.8**	**33.1**	**30.2**	**30.4**	**31.5**
**Total # of Studies 6/Subgroups 7**		**Total # of Joints**		**93**										
**Group 1 Total Means**			**14.4**		**45.1**	**25.9**	**23.0**	**20.3**	**25.2**	**56.2**	**29.6**	**28.7**	**27.1**	**28.7**
**Total # of Studies 34/Subgroups 45**		**Total Joints Group 1 & 2**		**1256**										

TNC: Total Nucleated Cells, E6= × 10^6^, E7= × 10^7^, E8= × 10^8.^

**Table 2:** Studies in Group 2, including outcomes, scores used and results.

Study	Cell Type	Study FU Length (Mo)	# of Joints	Clinical Outcome Tool Used	Improvement Scores	Results Summary
Al-Najar 2017 [32]	BMSCs	6	13	KOOS	Mean Subgroup improvements: Symptoms = 23.9, Pain = 26.49, ADLs = 26.61, Sport = 39.73, QoL = 41.33	Significant Improvement above MCID for all subgroups
Bastos 2018 [33]	BMSCs	12	9	KOOS	Mean Subgroup improvements: Symptoms = 19.2, Pain = 21.6, ADLs = 28.6, Sport = 24.3, QoL = 24.3	Significant Improvement above MCID for all subgroups
Bastos 2018 [33]	BMSCs + PRP	12	9	KOOS	Mean Subgroup improvements: Symptoms = 22.1, Pain = 32..9, ADLs = 31.4, Sport = 33.9, QoL = 25.9	Significant Improvement above MCID for all subgroups
Centeno 2011 [34]	BMSCs	11.3	135	Likert scale % improvement	Likert scale showed 62% of patients had over 50% relief, and 41% of patients had over 75 % relief.	Significant improvement in majority of patients
Gibbs 2015 [46]	SVF	12	7	KOOS	Mean Subgroup improvements (6 mo): Symptoms = 40.9, Pain = 37.4, ADLs = 33.9, Sports = 57.9, QOL = 49.1; (12 mo) Symptoms = 51.4, Pain = 43.0, ADL = 37.9, Sports = 64.3, QoL = 70.6	Significant Improvement above MCID for all subgroups out to 1 yr
Lamo-Espinosa 2018 low dose [39]	BMSCs	48	10	Median WOMAC & VAS	Median Improvement WOMAC 20 pts & VAS 50 pts	Significant improvement in WOMAC and VAS above MCID
Lamo-Espinosa 2018 high dose [39]	BMSCs	48	10	Median WOMAC & VAS	Median Improvement WOMAC 12.5 pts & VAS 30 pts	Significant improvement in WOMAC and VAS above MCID

**Table 3:** Group 3 studies—placebo arms of placebo-controlled trials of injectate treatments for knee osteoarthritis.

	WOMAC Peak Effect ^#^			VAS Peak Effect ^#^				WOMAC Scores					VAS Scores				
Author/Year/Cohort	Time (Wks)	Δ Score	Exceeds MCID?	Time (Wks)	Δ Score	Exceeds MCID?	# of Joints	Pre-treatment	3 MO	Δ 3 MO	6 MO	Δ 6 Mo	Pre-treatment	3 Mo	Δ 3 MO	6 MO	Δ 6 Mo
Altman 2004 [55]	12	13.2	Y	NA	NA	NA	174	46.9	33.7	13.2	35.8	11.1	NA	NA	NA	NA	NA
Altman 2009 [56]	*	*	Y	NA	NA	NA	259	NA	NA	NA	NA	14.4	NA	NA	NA	NA	NA
Baltzer 2009 [57]	7	13.0	Y	7	19.6	N	99	49.6	38.2	11.3	37.8	11.8	66.3	48.8	17.5	48.2	18.1
Bar-Or 2014–10 mL [58]	*	*	Y	NA	NA	NA	81	44.3	30.4	13.9	NA	NA	NA	NA	NA	NA	NA
Bar-Or 2014–4 mL [58]	*	*	Y	NA	NA	NA	83	42.6	29.0	13.6	NA	NA	NA	NA	NA	NA	NA
Chao 2010 [53]	4	1.0	N	NA	NA	NA	29	45.3	45.9	−0.6	NA	NA	NA	NA	NA	NA	NA
Chevalier 2010 [59]	*	*	Y	NA	NA	NA	129	54.6	NA	NA	42.4	12.2	NA	NA	NA	NA	NA
Henrotin 2017 [60]	NA	NA	NA	26	35.6	Y	41	NA	NA	NA	NA	NA	66.4	36.2	30.2	30.8	35.6
Karlsson 2002 [61]	12	18.2	Y	3	21.0	N	57	48.9	30.7	18.2	32.1	16.8	65.0	46.0	19.0	44.0	21.0
Kul-Panza 2010 [62]	5	7.9	N	14	23.0	Y	22	70.6	63.6	7.0	NA	NA	65.0	42.0	23.0	NA	NA
Lee 2015 [63]	12	7.0	N	12	14.0	N	27	37.0	30.0	7.0	30.0	7.0	64.0	50.0	14.0	52.0	12.0
Patel 2013 [54]	6	−1.2	N	6	0.0	N	46	45.5	50.7	−5.2	53.1	−7.6	45.7	NA	NA	46.1	−0.4
Ravaud 1999 [64]	NA	NA	NA	1	10.7	N	28	NA	NA	NA	NA	NA	63.7	61.2	2.5	58.2	5.5
Shrestha 2018 [65]	6	14.8	Y	2	10.3	N	58	56.5	56.1	0.4	NA	NA	67.3	69.0	-1.7	NA	NA
Smith 2016 [52]	8	15	Y	NA	NA	NA	15	46	37	9	44	2	NA	NA	NA	NA	NA
Takamura 2018 [66]	*	*	Y	NA	NA	NA	535	NA	NA	NA	NA	19.6	NA	NA	NA	NA	NA
Van der Weegen 2015 [67]	12	16.5	Y	12	9.8	N	97	40.8	22.5	16.5	28.8	12.0	24.6	14.8	9.8	21.5	3.1
Wu 2018 [68]	26	16.1	Y	NA	NA	NA	20	28.8	13.4	14.8	12.2	16.1	NA	NA	NA	NA	NA
Yavuz 2011 [69]	NA	NA	NA	1	15.0	N	30	NA	NA	NA	NA	NA	76.0	74.0	2.0	NA	NA
**Mean Scores**	**10.0**	**11.0**		**8.4**	**15.9**			**47.0**	**37.0**	**9.2**	**35.1**	**10.5**	**60.4**	**49.1**	**12.9**	**43.0**	**13.6**
**Total # Studies 18/Arms 19**					**# of Patients**		**1793**										

* Only 1 endpoint reported, ^#^ Only includes studies that reported two or more follow-up time points.

**Table 4:** MCID ratios.

Author/Year/Cohort	Cell Type	Total MSC/TNC Dosage (Millions) ^#^	Study FU Length (Mo)	# of Joints	Δ 6 mo	MCID Ratio 6 mo	Δ Final FU	MCID Ratio Final
Freitag 2019 1 inj [25]	ASCs	100	12	9	22.5	2.0	23.4	2.1
Freitag 2019 2 inj [25]	ASCs	200	12	10	17.1	1.6	31.6	2.9
Jo 2017 low dose [26]	ASCs	100	24	3	18.0	1.6	26.3	2.4
Jo 2017 medium dose [26]	ASCs	50	24	3	20.5	1.9	43.9	4.0
Jo 2017 high dose [26]	ASCs	10	24	12	21.4	1.9	35.2	3.2
Lee-2019 [27]	ASCs	100	6	12	33.3	3.0	33.3	3.0
Lu-2019 [28]	ASCs	100	12	46	9.1	0.8	9.5	0.9
Pers 2016 low dose [29]	ASCs	2	6	6	33.2	3.0	33.2	3.0
Pers 2016 medium dose [29]	ASCs	10	6	6	19.1	1.7	19.1	1.7
Pers 2016 high dose [29]	ASCs	50	6	6	21.0	1.9	21.0	1.9
Song 2018 [30]	ASCs	20	24	14	36.8	3.3	22.4	2.0
**ASCs Means**			**14.2**		**22.9**	**2.1**	**27.2**	**2.5**
		**Total # of Joints**		**127**				
Al-Najar 2017 * [32]	BMSCs	30.5	6	13	31.6	3.2	31.6	3.2
Bastos 2018 MSCs * [33]	BMSCs	40	12	9	NA	NA	23.6	2.4
Bastos 2018 MSCs + PRP * [33]	BMSCs + PRP	40	12	9	NA	NA	29.2	2.9
Emadedin 2012 [36]	BMSCs	22	12	6	25.1	2.3	23.5	2.1
Emadedin 2015 [37]	BMSCs	31	30	6	26.1	2.4	28.1	2.6
Emadedin 2018 [38]	BMSCs	40	6	19	25.7	2.3	25.7	2.3
Lamo-Espinosa 2018 low dose [39]	BMSCs	10	48	10	NA	NA	20.0	1.8
Lamo-Espinosa 2018 high dose [39]	BMSCs	100	48	10	NA	NA	12.5	1.1
Orozco 2013/14 [40,41]	BMSCs	40	24	12	NA	NA	11.5	1.0
Soler 2016 [42]	BMSCs	40	12	15	NA	NA	17.5	1.6
**BMSCs Means**			**21.0**		**27.1**	**2.5**	**22.3**	**2.1**
		**Total # of Joints**		**109**				
Bansal 2017	SVF	100	24	10	18.0	1.6	23.0	2.1
Fodor 2016	SVF	14.1	12	8	NA	NA	32.5	3.0
Garza 2020 low dose	SVF	15	12	13	31.2	2.8	33.1	3.0
Garza 2020 high dose	SVF	30	12	13	26.0	2.4	32.5	3.0
Gibbs 2015 *	SVF	30.8	12	7	43.8	4.4	53.4	5.3
Yokota 2017	SVF	30	6	26	15.8	1.4	15.8	1.4
**SVF Means**			**12.9**		**27.0**	**2.5**	**31.7**	**3.0**
		**Total # of Joints**		**93**				
**Total Means**			**15.5**		**24.2**	**2.2**	**25.4**	**2.4**
			**Total**	**1256**				

* MCID ratio based on KOOS scores; **^#^** Millions of cells reported as MSC for ASCs and BMSCs, TNCs for SVF.
